# Dual-organ invasion is associated with a lower survival rate than single-organ invasion in distal bile duct cancer: A multicenter study

**DOI:** 10.1038/s41598-018-29205-z

**Published:** 2018-07-17

**Authors:** Kyueng-Whan Min, Dong-Hoon Kim, Byoung Kwan Son, Kyoung Min Moon, Eun-Kyung Kim, Young-Ha Oh, Mi Jung Kwon, Ho Soon Choi

**Affiliations:** 1 Department of Pathology, Hanyang University College of Medicine, Seoul, Republic of Korea; 2 Departments of Pathology, Kangbuk Samsung Hospital, Sungkyunkwan University School of Medicine, Seoul, Republic of Korea; 3 Departments of Internal Medicine Eulji Hospital, Eulji University School of medicine, Seoul, Republic of Korea; 4 Department of Internal Medicine, Gangneung Asan Hospital, University of Ulsan College of Medicine, Gangneung, Republic of Korea; 5 Departments of Pathology, Eulji Hospital, Eulji University School of medicine, Seoul, Republic of Korea; 6 Department of Pathology, Hallym University Sacred Heart Hospital, Hallym University College of Medicine, Anyang, Republic of Korea; 7 Department of Internal Medicine, Hanyang University College of Medicine, Seoul, Republic of Korea

## Abstract

The revised criteria of the 8^th^ American Joint Committee on Cancer (AJCC) cancer staging system consider depth of invasion as one of the factors that determine stage in distal bile duct (DBD) cancer, but exclude adjacent organ invasion. The aims were to evaluate the association between adjacent organ invasion and relapse-free survival (RFS) and overall survival (OS) after curative surgical resection of DBD cancer and to propose optimal criteria for predicting clinical outcomes. In this retrospective cohort study, 378 patients with DBD cancer treated in multi-institutions between 1996 and 2013 were investigated. This study evaluated the relationship between clinicopathologic parameters and adjacent organ invasion and used organ invasion to compare the survival times of each group. Among 204 patients with adjacent organ invasion, 152 were in the single-organ invasion group and 52 were in the dual-organ invasion group based on a review of microscopic slides. In univariate and multivariate analyses, patients with dual-organ invasion had a shorter RFS and OS time than those with single-organ invasion. Organ invasion should be included as one of the factors that determine the AJCC stage; this might ultimately help to predict better the survival rate of patients with DBD cancer.

## Introduction

Beginning in the 7^th^ American Joint Committee on Cancer (AJCC) staging manual, distal bile duct (DBD) cancer and perihilar bile duct (PBD) cancer were staged separately based on biological behaviors. The stages remain separated in the new, 8^th^ AJCC staging manual. The 8^th^ AJCC staging manual adopts different T criteria for PBD and DBD cancer. In the revised criteria, the T stage for DBD cancer is based on depth of invasion (DOI): T1, <5 mm; T2, 5–12 mm; T3, >12 mm^1^. In addition, N stage is classified according to the number of metastatic lymph nodes: N0, no metastatic regional lymph nodes; N1, 1–3 metastatic regional lymph nodes; N2, >3 metastatic regional lymph nodes^2^. One of the main changes in the 8^th^ AJCC staging manual is the exclusion of tumour infiltration of adjacent organs such as the pancreas, duodenum, and gallbladder. In DBD, a unique, complex anatomy comprising various organs could provide very important information to classify AJCC stage. To define tumour stage, the previous 7th AJCC staging system considered the association between the tumour and surrounding organs, which have particular, complex anatomic structures. The 8^th^ AJCC staging system referred to the millimeter-based criteria in patients collected from a single institution, but it was modified without considering the anatomical specificity^3^. Furthermore, cut- off value was determined in patients with both DBD or PBD cancer and did not take into account the different biological characteristics between DBD and PBD cancer. With the exception of adjacent organ invasion, controversy remains regarding other aspects of the 8th AJCC tumour staging as prognostic predictors in patients with DBD cancer.

The AJCC staging system according to adjacent organ invasion is still practically used in various cancers. Even in PBD cancer, criteria based on adjacent structures is still being used to determine the 8^th^ AJCC staging. The adjacent organ invasion might still provide important information for determining advanced-stage DBD cancer with successful tumour removal, although it was excluded from the T criteria, which only consider DOI. In the previous 7^th^ T criteria of the DBD cancer, the T1 and T2 stages were defined as “Tumour confined to the bile duct” and “tumour invading beyond the wall of the bile duct”, respectively, whereas the T3 stage included organ invasion, such as the gallbladder, pancreas, duodenum, or other adjacent organs. Nevertheless, an inadequate number of validation studies was the main reason that organ invasion was excluded from the 8^th^ AJCC staging system. Previously published data were reported based on the results of analyses of both DBD and PBD cancer, because both cancers were categorized as “extrahepatic bile duct cancer” until the 6^th^ edition of the AJCC staging manual^1,4–8^. Past studies on the relationship between patient survival and organ invasion had inadequate numbers of study participants, which is why they have little prognostic significance^9,10^.

We validated the prognostic impact of the 8^th^ and 7^th^ AJCC staging system in 374 patients with DBD cancer. We investigated the survival rate according to the number of infiltrating organ to further enhance prognostic accuracy. The aims of this study were to evaluate the association between adjacent organ invasion and relapse-free survival (RFS) and overall survival (OS) after curative surgical resection of DBD cancer and to suggest supplementary criteria for predicting clinical outcomes.

## Results

### Clinicopathological Characteristics

**Figure 1:**
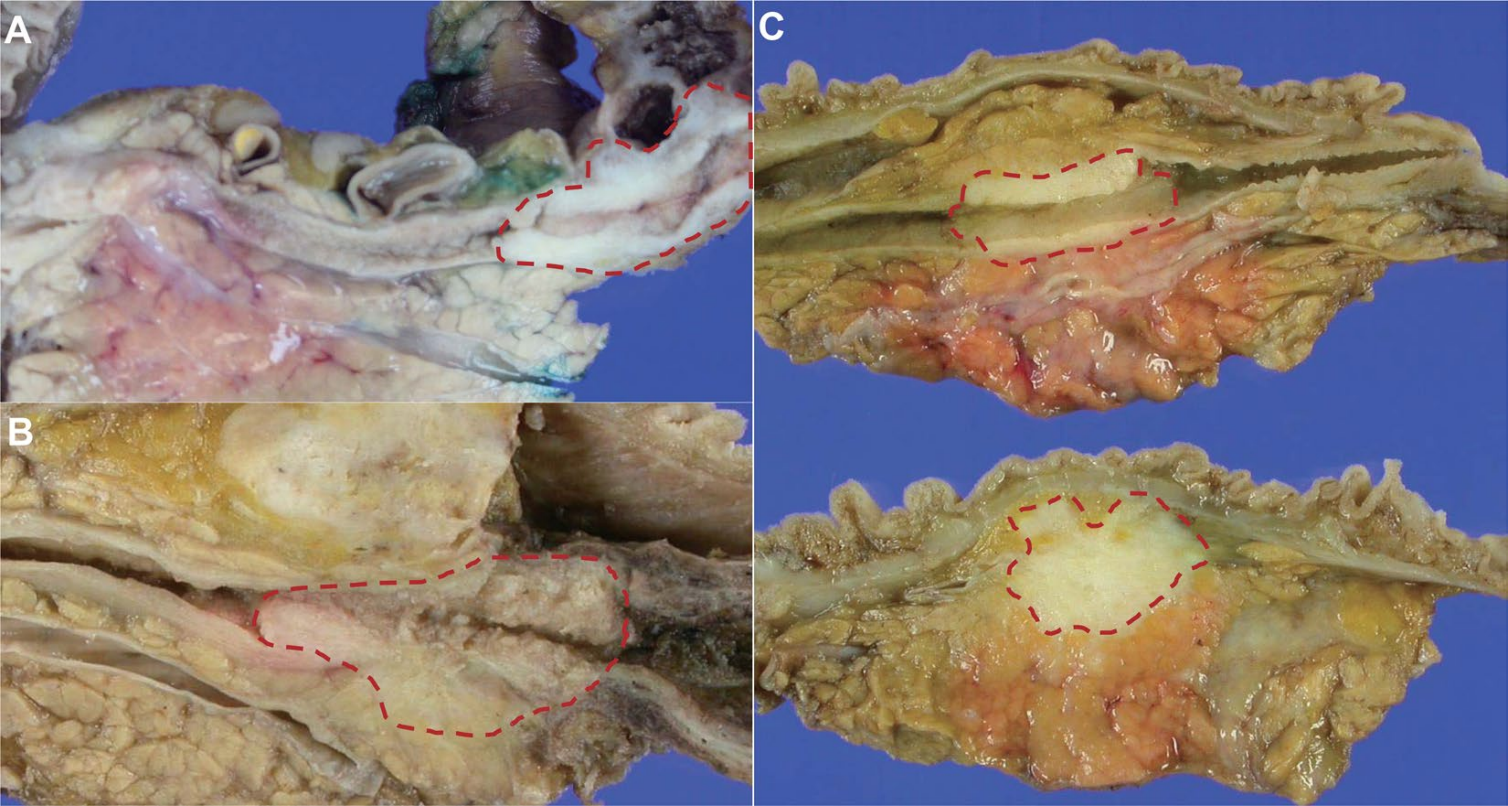
Gross view of distal bile duct cancer (red dashed circle): Tumour is confined to the bile duct wall without adjacent organ invasion (**A**). Tumour invades single organ (pancreas) (**B**). Tumour invades dual organs (pancreas and duodenum) (**C**).

At the time of surgery, the patients had the following T and N criteria: T1, 142 (37.6%); T2, 186 (49.2%); and T3, 50 (13.2%); N0, 196 (51.9%); N1, 154 (40.7%); N2, 28 (7.4%). Two hundred four patients had associated adjacent organ invasion, including invasion of the pancreas, duodenum, and gallbladder. One hundred fifty-two and 52 patients had single- and dual-organ invasion, respectively (Fig. 1). The distribution of AJCC stage was as follows: I, 94 (24.9%); IIA, 136 (36%); IIB, 120 (31.7%); and IIIA 28 (7.4%). Among 378 patients, 262 (69.3%) died during the follow-up period (median survival, 28 months; range, 4–195 months). Two hundred thirty (60.8%) had recurrence: 152 (40.2%) patients with local recurrence and 78 (20.6%) patients with new lymph node metastasis or new distant organ metastasis.

### Comparisons of Survival Rate According to Clinicopathological Parameters

**Figure 2:**
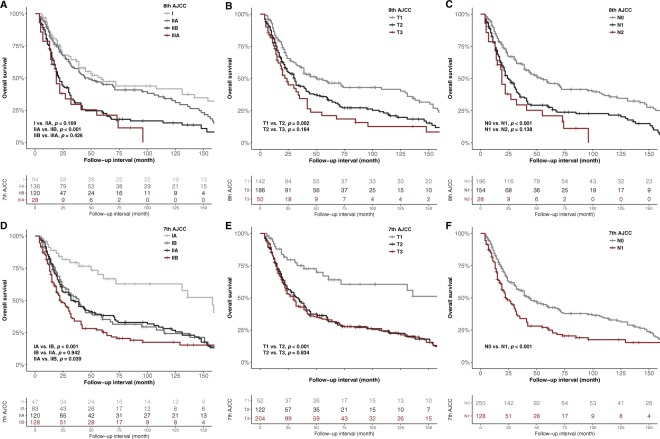
Kaplan–Meier survival curve according to the 8^th^ and 7^th^ AJCC staging system and T and N criteria. According to 8^th^ AJCC (**A**–**C**), the survival curve shows no survival difference between AJCC stage I versus IIA, AJCC stage IIB versus III, T2 versus T3 and N1 versus N2 (*p* = 0.169, 0.426, 0.164 and 0.138 respectively). According to 7^th^ AJCC (**D**–**F**), the survival curve shows no survival difference between AJCC stage IB versus IIA and T2 versus T3 (*p* = 0.942 and 0.834, respectively). There are relative differences of survival among the remaining groups.

Of 378 patients, the OS rates were as follow: 312 patients (82.5%) at 1 year, 197 (52.1%) at 3 years, and 164 (43.4%) at 5 years. The survival rate was lower with higher AJCC stages. However, there was no survival difference between stage I and IIA, between stage IIB and IIIA, between T2 and T3 or between N1 and N2, according to the AJCC stage. According to 7th AJCC stage, there was no survival difference between stage IB and IIA, between T2 and T3 (Fig. 2).

Other clinicopathological parameters such as gross type (HR, 1.67; 95% CI, 1.21–2.32; *P* = 0.002), histological grade (HR, 2; 95% CI, 1.48–2.7; *P* < 0.001), pancreatic invasion (HR, 1.33; 95% CI, 1.04–1.7; *P* = 0.021), duodenal invasion (HR, 1.94; 95% CI, 1.41–2.67; *P* < 0.001) lymphovascular invasion (HR, 1.42; 95% CI, 1.11–1.81; *P* = 0.005), perineural invasion (HR, 1.72; 95% CI, 1.3–2.27; *P* < 0.001), and margin status (HR, 1.42; 95% CI, 1.07–1.89; *P* = 0.015) were significantly related to OS. (Table 1)

**Table 1:** Clinicopathological characteristics and overall survival rate in 378 patients with distal bile duct cancer.

Parameters	N = 378	OS rate (%)			MST (95% CI)	
		1-year	3-year	5-year	Month	*p* value^*^
Age						
<65 y	196	84.2	56.6	45.4	30	0.101
≥65 y	182	80.8	47.3	41.2	22	
Sex						
Male	253	81.8	51	41.9	30	0.698
Female	125	84	54.4	46.4	22	
Gross type						
Papillary	25	88	68	56	39	**0.002** ^†^
Nodular	56	87.5	62.5	55.4	33	
Infiltrative	297	81.1	48.8	40.1	25	
Histological grade						
Well	81	91.4	71.6	56.8	50	**<0.001** ^‡^
Moderate	229	83.8	51.1	43.2	27	
Poor	68	67.6	32.4	27.9	16	
AJCC stage						
I	94	87.2	66	54.3	47	0.169^§^
IIA	136	86	61	51.5	32	
IIB	120	75.8	35.8	30	20	0.426^§^
IIIA	28	78.6	32.1	25	19	
T criteria						
1	142	86.6	61.3	52.1	43	0.164^¶^
2	186	81.2	47.8	39.8	24	
3	50	76	42	32	19	
N criteria						
0	196	86.7	64.3	53.1	39	0.138^‖^
1	154	77.9	40.3	34.4	22	
2	28	78.6	32.1	25	19	
Size (cm)						
<2.5	184	84.2	56	47.3	33	0.111
≥2.5	194	80.9	48.5	39.7	24	
Pancreas invasion						
Absence	181	84	59.1	49.7	35	**0.021**
Presence	197	81.2	45.7	37.6	22	
Duodenal invasion						
Absence	322	84.8	55.3	46.6	30	**<0.001**
Presence	56	69.6	33.9	25	19	
Gallbladder invasion						
Absence	375	82.4	52	43.7	27	0.43
Presence	3	100	66.7	—	47	
Lymphatic invasion						
Not identified	225	86.7	60	50.2	34	**0.005**
Present	153	76.5	40.5	33.3	21	
Perineural invasion						
Not identified	119	88.2	66.4	60.5	42	**<0.001**
Present	259	79.9	45.6	35.5	24	
Margin involvement						
Not involved	287	84.7	55.1	45.3	31	**0.015**
Involved	91	75.8	42.9	37.4	21	

AJCC, 8^th^ edition of American Joint Committee on Cancer; OS, overall survival; MST, median survival time.

^*^Log Rank test.

^†^papillary and nodular versus infiltrative type.

^‡^Well and moderately versus poorly differentiated.

^§^I versus IIA IIB versus IIIA.

^¶^T2 versus T3.

^‖^N1 versus N2.

*P-values* < 0.05 are in bold.

### Comparisons of Clinicopathological Parameters in Patients with and without Organ Involvement

Of all 378 patients, 204 patients had adjacent organ invasion. Patients with organ invasion had a significantly higher incidence of infiltrative gross type, poorly histological grade, lymphovascular invasion, perineural invasion and margin involvement, compared to those without organ invasion (all *P* < 0.05) (Table 2).

**Table 2:** Clinicopathological difference in patients with and without organ involvement in 378 patients with distal bile duct cancer.

Parameters	N = 378	Adjacent organ invasion		*p*-value (χ^2^ test)
		no organ invasion (n = 174), %	organ invasion (n = 204), %	
Age				
<65 y	196	83 (47.7)	113 (55.4)	0.136
≥65 y	182	91 (52.3)	91 (44.6)	
Sex				
Male	253	125 (71.8)	128 (62.7)	0.061
Female	125	49 (28.2)	76 (37.3)	
Gross type				
Papillary	25	18 (10.3)	7 (3.4)	**<0.001** ^*^
Nodular	56	35 (20.1)	21 (10.3)	
Infiltrative	297	121 (69.5)	176 (86.3)	
Histological grade				
Well	81	48 (27.6)	33 (16.2)	**0.003** ^*^
Moderate	229	102 (58.6)	127 (62.3)	
Poor	68	24 (13.8)	44 (21.6)	
N criteria				
0	196	96 (55.2)	100 (49)	0.08^*^
1	154	70 (40.2)	84 (41.2)	
2	28	8 (4.6)	20 (9.8)	
Size (cm)				
<2.5	184	92 (52.9)	92 (45.1)	0.132
≥2.5	194	82 (47.1)	112 (54.9)	
Lymphatic invasion				
Not identified	225	118 (67.8)	107 (52.5)	**0.002**
Present	153	56 (32.2)	97 (47.5)	
Perineural invasion				
Not identified	119	66 (37.9)	53 (26)	**0.013**
Present	259	108 (62.1)	151 (74)	
Margin involvement				
Not involved	287	109 (62.6)	178 (87.3)	**<0.001**
Involved	91	65 (37.4)	26 (12.7)	

^*^Linear-by-linear association.

*P-values* < 0.05 are in bold.

### Comparisons of Clinicopathological Parameters Between Single- and Dual-organ Invasion

Of 204 patients with adjacent organ invasion, 152 patients had adjacent single-organ invasion as follows: pancreas, 146; duodenum, 4; gallbladder, 2. Fifty-two patients had dual-organ invasion as follows: pancreas and duodenum, 51; duodenum and gallbladder, 1. Patients with dual-organ invasion had a significantly higher incidence of infiltrative gross type and advanced N stage, compared to those with single-organ invasion (all *P* < 0.05) (Table 3).

**Table 3:** Clinicopathological difference between single- and dual-organ involvement in 204 patients with distal bile duct cancer.

Parameters	N = 204	Adjacent organ invasion		*p*-value (χ^2^ test)
		single (n = 152), %	dual (n = 52), %	
Age				
<65 y	113	83 (54.6)	30 (57.7)	0.699
≥65 y	91	69 (45.4)	22 (42.3)	
Sex				
Male	128	93 (61.2)	35 (67.3)	0.43
Female	76	59 (38.8)	17 (32.7)	
Gross type				
Papillary	7	7 (4.6)	0 (0)	**0.039** ^*^
Nodular	21	18 (11.8)	3 (5.8)	
Infiltrative	176	127 (83.6)	49 (94.2)	
Histological grade				
Well	33	27 (17.8)	6 (11.5)	0.565^*^
Moderate	127	92 (60.5)	35 (67.3)	
Poor	44	33 (21.7)	11 (21.2)	
N criteria				
0	100	83 (54.6)	17 (32.7)	**0.022** ^*^
1	84	55 (36.2)	29 (55.8)	
2	20	14 (9.2)	6 (11.5)	
Size (cm)				
<2.5	92	70 (46.1)	22 (42.3)	0.639
≥2.5	112	82 (53.9)	30 (57.7)	
Lymphatic invasion				
Not identified	107	85 (55.9)	22 (42.3)	0.09
Present	97	67 (44.1)	30 (57.7)	
Perineural invasion				
Not identified	53	39 (25.1)	14 (26.9)	0.857
Present	151	113 (74.3)	38 (73.1)	
Margin involvement				
Not involved	178	129 (84.9)	49 (94.2)	0.081
Involved	26	23 (15.1)	3 (5.8)	

^*^Linear-by-linear association

*P-values* < 0.05 are in bold.

### Survival Difference Between Single- and Dual-Organ Invasion in 204 Patients

The RFS time was as follows: For patients with no organ invasion, the median survival was 27 months (recurrent rate: 51.7%, 90/174), for single-organ invasion, the median survival was 23 months (66.4% 101/152), and for dual-organ invasion, the median survival was 13 months (75%, 39/52). The OS time was as follows: For patients with no organ invasion, the median survival was 35 months (mortality rate: 61.5%, 107/174), for single-organ invasion, the median survival was 29 months (73%, 111/152) and for dual-organ invasion, the median survival was 19 months (84.6%, 44/52).

**Figure 3:**
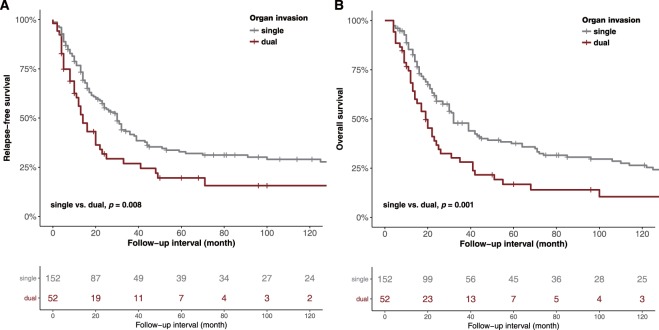
Kaplan–Meier survival analysis stratified according to organ invasion. Patients with single-organ invasion have a lower relapse-free and overall survival rate than those with dual-organ invasion (*p* = 0.008 and 0.001).

In univariate analyses of 202 patients with organ invasion, the RFS (HR, 1.65; 95% CI, 1.14–2.4; *P* = 0.008) and OS (HR, 1.87; 95% CI, 1.31–2.66; *P* = 0.001) were significantly different between patients with single- and dual-organ invasion (Fig. 3). Other factors including histological grade (RFS: HR, 1.89; 95% CI, 1.28–2.77; *P* = 0.001; OS: HR, 1.73; 95% CI, 1.19–2.52; *P* = 0.004) and N criteria (RFS: HR, 1.71; 95% CI, 1.22–2.39; *P* = 0.002; OS: HR, 1.59; 95% CI, 1.15–2.19; *P* = 0.005) were also associated with a worse RFS and OS. In multivariate analysis (confounding factors: gross type histological grade, T criteria, N criteria, lymphatic/perineural invasion and margin status), there was a significant difference in RFS (HR, 1.67; 95% CI, 1.12–2.49; *P* = 0.013) and OS (HR, 1.95; 95% CI, 1.32–2.87; *P* = 0.001), between patients with single- and dual-organ invasion. In addition, higher histological grade (RFS: HR, 2.25; 95% CI, 1.5–3.39; *P* < 0.001; OS: HR, 2; 95% CI, 1.32–2.92; *P* = 0.001) and advanced N stage (RFS: HR, 1.73; 95% CI, 1.18–2.53; *P* = 0.005; OS: HR, 1.47; 95% CI, 1.03–2.1; *P* = 0.036) remained associated with poor RFS and OS (Table 4).

**Table 4:** Survival difference between single- and dual-organ invasion in 204 patients with distal bile duct cancer.

Survival	Univariate significance^*^	Multivariate significance^†^	HR	95% CI	
				Low	Upper
Relapse-free survival					
Organ invasion (single vs. dual)	**0.008**	**0.013**	1.668	1.116	2.492
Gross type (papillary or nodular *vs*. infiltrative)	0.995	0.402	0.802	0.478	1.344
Histological grade (well or moderate vs. poor)	**0.001**	**<0.001**	2.251	1.493	3.393
T criteria (1 or 2 vs. 3)	0.239	0.787	1.060	0.694	1.620
N criteria (0 vs. 1 or 2)	**0.002**	**0.005**	1.732	1.184	2.533
Lymphatic invasion (absence *vs*. presence)	0.409	0.174	0.771	0.531	1.122
Perineural invasion (absence *vs*. presence)	0.115	0.138	1.363	0.905	2.052
Margin involvement (negative *vs*. positive)	0.25	0.383	1.246	0.760	2.042
Overall survival					
Organ invasion (single vs. dual)	**0.001**	**0.001**	1.946	1.322	2.865
Gross type (papillary or nodular *vs*. infiltrative)	0.93	0.317	0.769	0.460	1.286
Histological grade (well or moderate vs. poor)	**0.004**	**0.001**	1.959	1.316	2.916
T criteria (1 or 2 vs. 3)	0.199	0.983	1.004	0.671	1.503
N criteria (0 vs. 1 or 2)	**0.005**	**0.036**	1.468	1.025	2.102
Lymphatic invasion (absence *vs*. presence)	0.161	0.642	0.919	0.644	1.311
Perineural invasion (absence *vs*. presence)	0.084	0.175	1.312	0.886	1.942
Margin involvement (negative *vs*. positive)	0.168	0.154	1.438	0.873	2.370

^*^Log Rank test.

^†^Cox proportional hazard model.

*P-values* < 0.05 are in bold.

## Discussion

In the new 8^th^ AJCC staging manual, cholangiocarcinoma is classified based on its anatomic location into three subtypes: intrahepatic, perihilar, and distal portion. Importantly, DBD cancers comprise 20–30% of all cholangiocarcinoma and are clinically silent, with symptoms only developing at an advanced stage^11^. Beginning in the 7^th^ edition, and continuing in the 8^th^ edition, the T and N criteria are different for PBD and DBD and are based on their distinct biological behavior, natural course, and therapeutic plan^11,12^. In particular, the factors that were specifically associated with patient survival were DOI, nodal metastasis, lymphatic/perineural invasion as well as pancreatic invasion, and resection margin involvement, to name a few^1,4–6^.

Interestingly, among the above prognostic indicators, DOI and number of metastatic regional lymph nodes were applied in the new 8^th^ T and N criteria for DBD cancer, because the previous 7^th^ AJCC staging was described as having vague T criteria resulting in wide inter-observer variation. Therefore, the 8^th^ AJCC staging system suggested a cut-off value of DOI measured in millimeters to reduce inter-observer variation^1,13,14^. To determine the stage of tumours in various organs (lip and oral cavity, cervix uteri, vulva, and melanoma of the skin), DOI was adopted in the 8^th^ AJCC staging system. These organs have simple anatomical structures and are relatively distant from tumour-adjacent organs. In other words, a long distance from the tumour origin inhibits direct tumour infiltration into other organs. However, the DBD is located near various organs and has a relatively complex anatomical structure. In organs that are close to tumour origins, such as the nasal cavity, paranasal sinus, and larynx, the T criteria are classified based on direct tumour infiltration into adjacent organs. A study of DBD cancer showed that the presence or absence of adjacent organ invasion was associated with a significant difference in survival^9^. Nevertheless, for DBD cancer, the 7^th^ AJCC staging manual categorizes T criteria based on adjacent organ invasion, but organ invasion is no longer described in the 8^th^ AJCC T criteria, especially T3 stage^3^. A study by Ebata *et al*. showed that presence or absence of adjacent organ invasion created a significant difference in survival^9^. In our results, patients with organ invasion show lower RFS and OS than those without organ invasion. Notably, there were significant differences of RFS or OS between single- and dual-organ invasion. However, there was little survival difference when the 8^th^ AJCC T criteria were adopted for DBD cancer. An explanation for this is that the interval of invasion depth among T1, T2, and T3 groups was widened. In our study, the categories of DOI were as follows: no invasion, 4.5 mm; single-organ invasion, 8.2 mm; dual-organ invasion, 10.7 mm.

The recommended 8^th^ AJCC T criteria for DOI are 5–12 mm and >12 mm in the T2 and T3 groups, respectively. Hong *et al*. performed a study of 222 patients who underwent surgery at a single center and whose tumours included both perihilar and/or distal tumours (perihilar, 111 cases; distal 101 cases; perihilar and distal, 10 cases)^3^. In survival models to determine the cut-off value of DOI, only 101 patients had DBD cancer, whereas 110 patients had PBD cancer. The cut-off values for the measured DOI were calculated in both perihilar and DBD cancers without considering their distinct biological behaviors^11,12^. In a validation study, there was no survival difference between groups (T2 versus T3)^15^. Recently, a multicenter study of 179 patients with only DBD cancer was designed using a smaller range of DOI and revealed a significant survival difference among groups (<3 mm, 4–10 mm, >11 mm)^13^. Thus, controversy exists regarding the relationship between clinical outcome and the criteria for DOI.

In summary, patients with dual-organ invasion showed lower survival rate than patients with single-organ invasion in DBD cancer, although there is no survival difference between the T2 and T3 groups based on DOI defined by the 8^th^ AJCC staging system. In the prognosis prediction with advanced T groups, adjacent organ invasion could enhance prognostic accuracy. Consequently, the significant difference in survival between single- and dual-organ invasion could be considered to supplement the T criteria using DOI to guide therapy and standardize the 8^th^ AJCC staging system.

## Materials and Methods

### Case Selection

Tumour with their center located between the confluence of the cystic duct and common hepatic duct and the Ampulla of Vater (excluding ampullary cancer) are considered DBD cancer in reference with 8^th^ AJCC stage. A total of 404 cases of patients diagnosed with DBD cancer at multi-institutions (Eulji Hospital, Kangbuk Samsung, Hanyang Guri, Hallym University Sacred Heart Hospital, Gangneung Asan) in Korea between January 1, 1996 and December 31, 2013 were collected for this study. This study included data obtained from previously conducted research study^13^. As for twenty-six patients who died within 90 days after surgery or who had few representative slide for microscopic review were excluded from this study.

The following clinicopathological parameters were recorded: age, gross type, histological grade, size, 8^th^ AJCC stage, lymph node metastasis, adjacent organ invasion (pancreas, duodenum, gallbladder), lymphovascular invasion, perineural invasion, margin involvement, relapse, and survival. Grossly, the tumours were classified as papillary, nodular, and infiltrative, and the tumour size was measured along its greatest dimension. Hematoxylin and eosin-stained slides with representative tumour section were reviewed by at three pathologists (KWM, DHK, EKK). The DOI from the basal lamina of the adjacent normal epithelium to the most deeply advanced tumour cells was measured in reference with previous study^3^.

The mean and median age of the remaining 378 patients was 63 and 64 years, respectively. The male to female ratio was 253:125. The surgical treatment included the Whipple procedure in 153 (40.5%), pylorus-preserving pancreaticoduodenectomy in 125 (33.1%), and extended bile duct resection in 101 (26.4%) patients. In multi-institutions, the indication of surgical procedure for DBD cancer was as follows: pylorus-preserving pancreaticoduodenectomy was performed on patients with (a) no evidence of tumour extension to the pylorus, (b) chances to preserve pylorus artery and (c) no ulcer in pylorus. Whipple’s operation was conducted when a patient did not belong to the above PPPD indication. Extended bile duct resection was conducted when (a) tumour was positioned at mid-portion and did not invade adjacent organs and (b) safety margin is confirmed by frozen section of tumour.

### Statistical Analysis

Correlations between clinicopathological parameters and adjacent organ invasion were analysed using the Chi-square test and the linear-by-linear association. Relapse-free survival is defined as the time elapsed from the date of treatment to the date of progression such as a local recurrence, new lymph node metastasis or distant organ metastasis. Overall survival was defined as the time from the date of treatment to cancer-related death. Relapse-free survival (RFS) and overall survival (OS) curves were generated using the Kaplan-Meier method and were compared using the Log Rank test. Multivariate analysis was performed to confirm independent prognostic factors for patient survival using a Cox proportional hazard model. A 2-tailed *P* value of <0.05 was considered statistically significant. All data were analysed using SPSS statistics software (version 20.0, Chicago, IL, USA) and R packages (http://www.r-project.org/).

### Ethics Approval

This study (involving human participants) was approved by the Ethics Committee of the Eulji Hospital (EMCIRB-2016-10-001) and performed with respect to the ethical standards of the Declaration of Helsinki, as revised in 2008. The IRB review confirmed that the informed consent is not necessary in this study.
