# Author Correction: Dual-organ invasion is associated with a lower survival rate than single-organ invasion in distal bile duct cancer: A multicenter study

**DOI:** 10.1038/s41598-018-30161-x

**Published:** 2018-08-10

**Authors:** Kyueng-Whan Min, Dong-Hoon Kim, Byoung Kwan Son, Kyoung Min Moon, Eun-Kyung Kim, Young-Ha Oh, Mi Jung Kwon, Ho Soon Choi

**Affiliations:** 1 Department of Pathology, Hanyang University College of Medicine, Seoul, Republic of Korea; 2 Departments of Pathology, Kangbuk Samsung Hospital, Sungkyunkwan University School of Medicine, Seoul, Republic of Korea; 3 Departments of Internal Medicine Eulji Hospital, Eulji University School of medicine, Seoul, Republic of Korea; 4 Department of Internal Medicine, Gangneung Asan Hospital, University of Ulsan College of Medicine, Gangneung, Republic of Korea; 5 Departments of Pathology, Eulji Hospital, Eulji University School of medicine, Seoul, Republic of Korea; 6 Department of Pathology, Hallym University Sacred Heart Hospital, Hallym University College of Medicine, Anyang, Republic of Korea; 7 Department of Internal Medicine, Hanyang University College of Medicine, Seoul, Republic of Korea

Correction to: *Scientific Reports* 10.1038/s41598-018-29205-z, published online 17 July 2018

The original version of this Article contained an error in the title of the paper, where the word “in” was omitted between “invasion” and “distal”. This has now been corrected in the PDF and HTML versions of the Article.

